# 4-(2-Hy­droxy­phen­yl)-3,5-di­thia­hepta­ne­dioic acid

**DOI:** 10.1107/S1600536813012737

**Published:** 2013-05-15

**Authors:** K. Ravichandran, R. Manikannan, S. Muthusubramanian, P. Ramesh, M. N. Ponnuswamy

**Affiliations:** a Department of Physics, Kandaswami Kandar’s College, Velur, Namakkal 638 182, India; b Institute of Organic Chemistry and Technology, Faculty of Chemical Technology, University of Pardubice, Studentska 573, 532 10 Pardubice, Czech Republic; c Department of Organic Chemistry, School of Chemistry, Madurai Kamaraj University, Madurai 625 021, India; d Centre of Advanced Study in Crystallography and Biophysics, University of Madras, Guindy Campus, Chennai 600 025, India

## Abstract

In the crystal of the title compound, C_11_H_12_O_5_S_2_, mol­ecules are linked by O—H⋯O hydrogen bonds and C—H⋯O inter­actions, forming a three-dimensional network.

## Related literature

For related structures, see: Guo *et al.* (2010 ▶); Yu *et al.* (2010 ▶); Rollas & Kucukguzel (2007 ▶). For bond-length data, see: Allen *et al.* (1987 ▶). For hydrogen-bond motifs, see: Bernstein *et al.* (1995 ▶). 
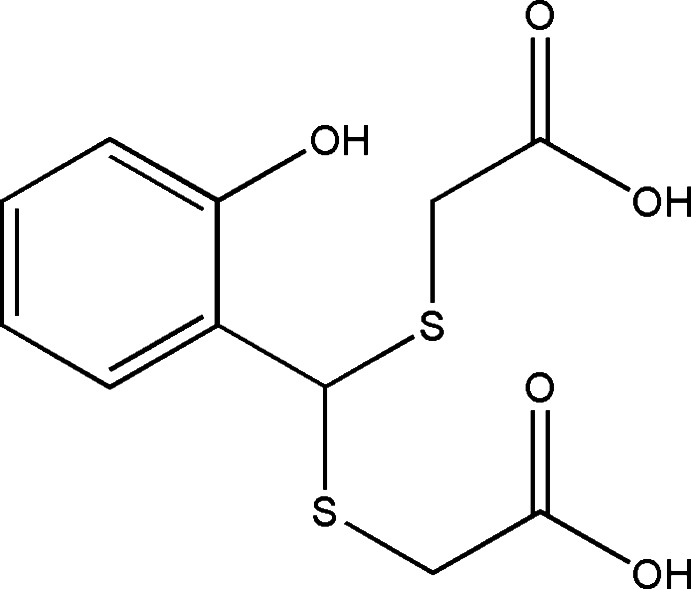


## Experimental

### 

#### Crystal data

C_11_H_12_O_5_S_2_
*M* *_r_* = 288.33
Triclinic, 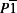

*a* = 7.2465 (1) Å
*b* = 7.6533 (1) Å
*c* = 12.0141 (2) Å
α = 101.094 (2)°
β = 99.129 (1)°
γ = 102.390 (2)°
*V* = 624.57 (2) Å^3^
*Z* = 2
Mo *K*α radiation
μ = 0.44 mm^−1^
*T* = 293 K
0.20 × 0.18 × 0.17 mm

#### Data collection

Bruker SMART APEXII CCD diffractometer
Absorption correction: multi-scan (*SADABS*; Bruker, 2008 ▶) *T*_min_ = 0.916, *T*_max_ = 0.929
2746 measured reflections
2180 independent reflections
1918 reflections with *I* > 2σ(*I*)
*R*_int_ = 0.008

#### Refinement

*R*[*F* ^2^ > 2σ(*F* ^2^)] = 0.026
*wR*(*F* ^2^) = 0.073
*S* = 1.06
2180 reflections
175 parameters
H atoms treated by a mixture of independent and constrained refinement
Δρ_max_ = 0.24 e Å^−3^
Δρ_min_ = −0.21 e Å^−3^

### 

Data collection: *APEX2* (Bruker, 2008 ▶); cell refinement: *SAINT* (Bruker, 2008 ▶); data reduction: *SAINT*; program(s) used to solve structure: *SHELXS97* (Sheldrick, 2008 ▶); program(s) used to refine structure: *SHELXL97* (Sheldrick, 2008 ▶); molecular graphics: *ORTEP-3* for Windows (Farrugia, 2012 ▶); software used to prepare material for publication: *SHELXL97* and *PLATON* (Spek, 2009 ▶).

## Supplementary Material

Crystal structure: contains datablock(s) global, I. DOI: 10.1107/S1600536813012737/bt6906sup1.cif

Structure factors: contains datablock(s) I. DOI: 10.1107/S1600536813012737/bt6906Isup2.hkl

Supplementary material file. DOI: 10.1107/S1600536813012737/bt6906Isup3.cml

Additional supplementary materials:  crystallographic information; 3D view; checkCIF report

## Figures and Tables

**Table 1:** Hydrogen-bond geometry (Å, °)

*D*—H⋯*A*	*D*—H	H⋯*A*	*D*⋯*A*	*D*—H⋯*A*
C2—H2⋯O3^i^	0.93	2.58	3.395 (2)	146
O1—H1⋯O4^ii^	0.75 (2)	1.97 (2)	2.7143 (19)	173 (3)
C8—H8*B*⋯O5^iii^	0.97	2.53	3.429 (2)	154
O2—H2*A*⋯O1^iv^	0.81 (3)	1.92 (3)	2.706 (2)	163 (2)
O5—H5*A*⋯O3^v^	0.79 (3)	1.97 (3)	2.7459 (19)	165 (3)

Symmetry codes: (i) 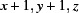; (ii) 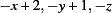; (iii) 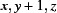; (iv) 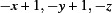; (v) 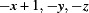.

**Fig. 1.**
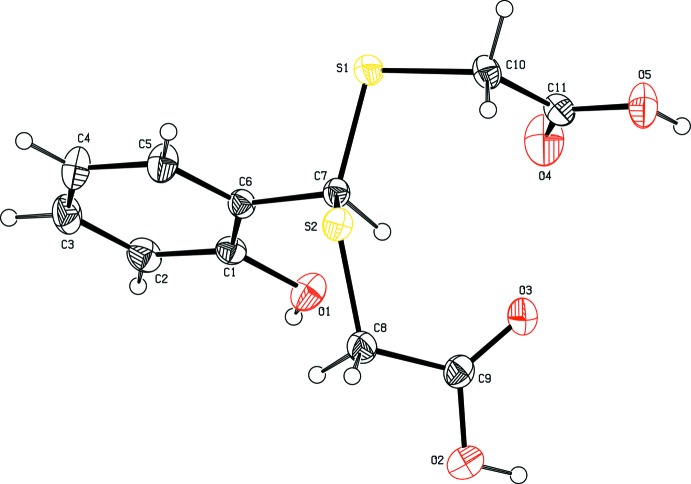
The molecular structure of the title compound, showing the atomic numbering and displacement ellipsoids drawn at the 30% probability level.

**Fig. 2.**
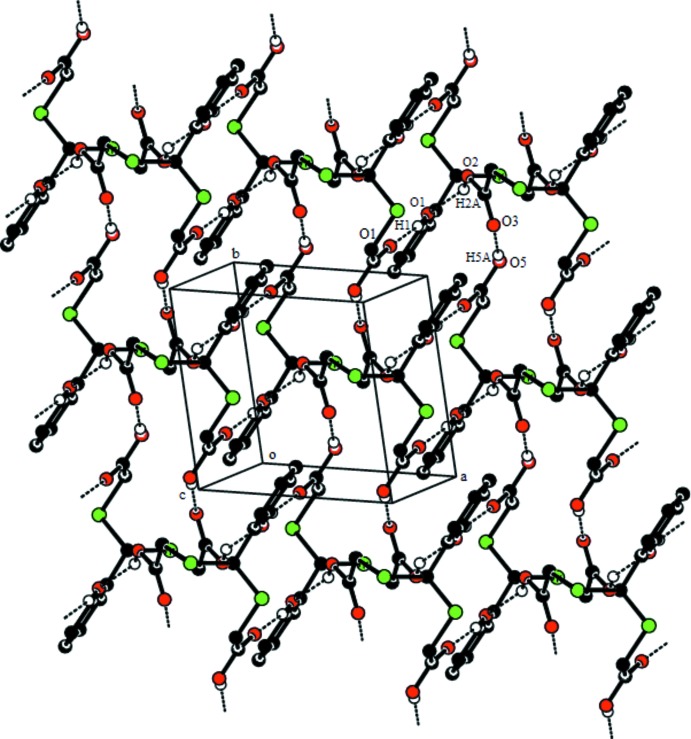
The packing of the molecules viewed down c axis.

## supplementary crystallographic information

### Comment

In coordination chemistry, the acylhydrazone ligands attracted the chemists due to their potential behaviour in magnetochemistry (Yu *et al.*, 2010; Guo *et al.*, 2010). The choice of *N*-acylhydrazonyl derivatives was suggested by publications indicating that compounds with such groups might have anti-tumoural activities (Rollas & Kucukguzel 2007). Against this background and to ascertain the molecular structure of title compound, the crystallographic study has been carried out.

The *ORTEP* plot of the molecule is shown in Fig. 1. The bond lengths involving the sulfur atoms in methyl sulfanyl groups [S1—C7=] 1.821 (2) Å, [S1—C10=] 1.799 (2) Å, [S2—C7=] 1.830 (2) Å and [S2—C8=] 1.794 (2) Å are comparable with the standard values of 1.82 Å reported in the literature (Allen *et al.*, 1987).

The crystal structure is stabilized by C—H···O and O—H···O types of intra and intermolecular interactions which form a three demensional network shown in Fig. 2. The intermolecular O1—H1···O4 and O2—H2A···O1 hydrogen bonds form two different *R*_2_^2^(18) dimers. The molecules at O5 (*x*, *y*, *z*) and O3 (-*x* + 1, -*y*, -*z*) are linked through an intermolecular O5—H5A···O3 hydrogen bond into cyclic centrosymmetric *R*_2_^2^(20) dimer (Bernstein *et al.*, 1995).

### Experimental

A mixture of salicylaldehyde and freshly distilled thioglycollic acid in 1:1.2 mole ratio was dissolved by heating on a water bath and let stand two days. The product was washed with water and recrystallized.

### Refinement

H atoms bonded to O were isotropically refined and the other H atoms were positioned geometrically (C—H=0.93–0.98 Å) and allowed to ride on their parent atoms, with 1.2 *U*_eq_(C).

### Crystal data

**Table:**

C_11_H_12_O_5_S_2_	*Z* = 2
*M**_r_* = 288.33	*F*(000) = 300
Triclinic, *P*1	*D*_x_ = 1.533 Mg m^−^^3^
Hall symbol: -P 1	Mo *K*α radiation, λ = 0.71073 Å
*a* = 7.2465 (1) Å	Cell parameters from 1918 reflections
*b* = 7.6533 (1) Å	θ = 2.8–25.0°
*c* = 12.0141 (2) Å	µ = 0.44 mm^−^^1^
α = 101.094 (2)°	*T* = 293 K
β = 99.129 (1)°	Block, white
γ = 102.390 (2)°	0.20 × 0.18 × 0.17 mm
*V* = 624.57 (2) Å^3^	

### Data collection

**Table:**

Bruker SMART APEXII CCD diffractometer	2180 independent reflections
Radiation source: fine-focus sealed tube	1918 reflections with *I* > 2σ(*I*)
Graphite monochromator	*R*_int_ = 0.008
ω and φ scans	θ_max_ = 25.0°, θ_min_ = 2.8°
Absorption correction: multi-scan (*SADABS*; Bruker, 2008)	*h* = −1→8
*T*_min_ = 0.916, *T*_max_ = 0.929	*k* = −9→9
2746 measured reflections	*l* = −14→14

### Refinement

**Table:**

Refinement on *F*^2^	Primary atom site location: structure-invariant direct methods
Least-squares matrix: full	Secondary atom site location: difference Fourier map
*R*[*F*^2^ > 2σ(*F*^2^)] = 0.026	Hydrogen site location: inferred from neighbouring sites
*wR*(*F*^2^) = 0.073	H atoms treated by a mixture of independent and constrained refinement
*S* = 1.06	*w* = 1/[σ^2^(*F*_o_^2^) + (0.0361*P*)^2^ + 0.2534*P*] where *P* = (*F*_o_^2^ + 2*F*_c_^2^)/3
2180 reflections	(Δ/σ)_max_ < 0.001
175 parameters	Δρ_max_ = 0.24 e Å^−^^3^
0 restraints	Δρ_min_ = −0.21 e Å^−^^3^

### Special details

**Table:**

Geometry. All e.s.d.'s (except the e.s.d. in the dihedral angle between two l.s. planes) are estimated using the full covariance matrix. The cell e.s.d.'s are taken into account individually in the estimation of e.s.d.'s in distances, angles and torsion angles; correlations between e.s.d.'s in cell parameters are only used when they are defined by crystal symmetry. An approximate (isotropic) treatment of cell e.s.d.'s is used for estimating e.s.d.'s involving l.s. planes.
Refinement. Refinement of *F*^2^ against ALL reflections. The weighted *R*-factor *wR* and goodness of fit *S* are based on *F*^2^, conventional *R*-factors *R* are based on *F*, with *F* set to zero for negative *F*^2^. The threshold expression of *F*^2^ > σ(*F*^2^) is used only for calculating *R*-factors(gt) *etc*. and is not relevant to the choice of reflections for refinement. *R*-factors based on *F*^2^ are statistically about twice as large as those based on *F*, and *R*- factors based on ALL data will be even larger.

### Fractional atomic coordinates and isotropic or equivalent isotropic displacement parameters (Å^2^)

**Table:**

	*x*	*y*	*z*	*U*_iso_*/*U*_eq_	
S1	1.00303 (6)	0.36671 (6)	0.31104 (4)	0.03515 (13)	
S2	0.67356 (6)	0.52625 (6)	0.35266 (4)	0.03286 (13)	
O1	0.9831 (2)	0.7027 (2)	0.09620 (11)	0.0426 (3)	
H1	1.038 (4)	0.747 (3)	0.057 (2)	0.056 (7)*	
O2	0.3103 (2)	0.5269 (2)	0.07080 (14)	0.0557 (4)	
H2A	0.241 (4)	0.450 (3)	0.016 (2)	0.059 (7)*	
O3	0.4267 (2)	0.29754 (18)	0.11941 (11)	0.0462 (3)	
O4	0.8498 (3)	0.1453 (2)	0.06126 (12)	0.0639 (5)	
O5	0.6463 (2)	−0.08304 (19)	0.10180 (13)	0.0520 (4)	
H5A	0.628 (4)	−0.128 (3)	0.035 (2)	0.066 (8)*	
C1	1.0698 (2)	0.7921 (2)	0.20917 (14)	0.0303 (4)	
C2	1.2097 (3)	0.9569 (2)	0.23501 (17)	0.0395 (4)	
H2	1.2477	1.0076	0.1753	0.047*	
C3	1.2930 (3)	1.0461 (3)	0.34859 (18)	0.0443 (5)	
H3	1.3869	1.1569	0.3655	0.053*	
C4	1.2370 (3)	0.9711 (3)	0.43746 (17)	0.0476 (5)	
H4	1.2925	1.0313	0.5143	0.057*	
C5	1.0979 (3)	0.8059 (3)	0.41159 (16)	0.0411 (4)	
H5	1.0612	0.7557	0.4717	0.049*	
C6	1.0119 (2)	0.7133 (2)	0.29760 (14)	0.0281 (3)	
C7	0.8675 (2)	0.5283 (2)	0.27182 (14)	0.0278 (3)	
H7	0.8109	0.4901	0.1885	0.033*	
C8	0.5047 (2)	0.5942 (2)	0.25420 (16)	0.0373 (4)	
H8A	0.4038	0.6229	0.2932	0.045*	
H8B	0.5710	0.7063	0.2362	0.045*	
C9	0.4115 (2)	0.4540 (2)	0.14220 (16)	0.0354 (4)	
C10	0.8202 (3)	0.1531 (2)	0.25863 (14)	0.0335 (4)	
H10A	0.8600	0.0627	0.2971	0.040*	
H10B	0.7017	0.1714	0.2811	0.040*	
C11	0.7775 (3)	0.0751 (2)	0.12984 (15)	0.0357 (4)	

### Atomic displacement parameters (Å^2^)

**Table:**

	*U*^11^	*U*^22^	*U*^33^	*U*^12^	*U*^13^	*U*^23^
S1	0.0314 (2)	0.0288 (2)	0.0430 (3)	0.00619 (17)	0.00384 (18)	0.00810 (18)
S2	0.0303 (2)	0.0346 (2)	0.0324 (2)	0.00405 (17)	0.00923 (17)	0.00719 (17)
O1	0.0395 (7)	0.0544 (8)	0.0304 (7)	0.0001 (6)	0.0077 (6)	0.0144 (6)
O2	0.0534 (9)	0.0514 (9)	0.0531 (9)	0.0128 (7)	−0.0113 (7)	0.0093 (7)
O3	0.0524 (8)	0.0349 (7)	0.0429 (7)	0.0075 (6)	0.0017 (6)	0.0002 (6)
O4	0.0885 (12)	0.0598 (9)	0.0378 (8)	−0.0036 (8)	0.0236 (8)	0.0149 (7)
O5	0.0655 (10)	0.0373 (8)	0.0396 (8)	−0.0047 (7)	0.0083 (7)	−0.0009 (6)
C1	0.0290 (8)	0.0311 (8)	0.0333 (9)	0.0102 (7)	0.0081 (7)	0.0091 (7)
C2	0.0379 (10)	0.0351 (9)	0.0494 (11)	0.0060 (8)	0.0153 (8)	0.0174 (8)
C3	0.0370 (10)	0.0299 (9)	0.0593 (12)	−0.0030 (8)	0.0113 (9)	0.0062 (8)
C4	0.0450 (11)	0.0421 (11)	0.0408 (10)	−0.0055 (9)	0.0040 (9)	−0.0027 (8)
C5	0.0449 (10)	0.0406 (10)	0.0315 (9)	−0.0016 (8)	0.0081 (8)	0.0070 (8)
C6	0.0253 (8)	0.0258 (8)	0.0324 (8)	0.0047 (6)	0.0067 (6)	0.0065 (6)
C7	0.0283 (8)	0.0261 (8)	0.0276 (8)	0.0039 (7)	0.0059 (6)	0.0063 (6)
C8	0.0313 (9)	0.0318 (9)	0.0459 (10)	0.0087 (7)	0.0050 (8)	0.0038 (8)
C9	0.0272 (8)	0.0358 (10)	0.0405 (9)	0.0028 (7)	0.0070 (7)	0.0086 (8)
C10	0.0400 (10)	0.0255 (8)	0.0346 (9)	0.0043 (7)	0.0115 (7)	0.0079 (7)
C11	0.0422 (10)	0.0296 (9)	0.0369 (9)	0.0107 (8)	0.0103 (8)	0.0085 (7)

### Geometric parameters (Å, º)

**Table:**

S1—C10	1.7984 (17)	C2—H2	0.9300
S1—C7	1.8207 (16)	C3—C4	1.382 (3)
S2—C8	1.7940 (18)	C3—H3	0.9300
S2—C7	1.8296 (16)	C4—C5	1.383 (3)
O1—C1	1.376 (2)	C4—H4	0.9300
O1—H1	0.75 (2)	C5—C6	1.390 (2)
O2—C9	1.321 (2)	C5—H5	0.9300
O2—H2A	0.81 (3)	C6—C7	1.513 (2)
O3—C9	1.209 (2)	C7—H7	0.9800
O4—C11	1.195 (2)	C8—C9	1.504 (2)
O5—C11	1.315 (2)	C8—H8A	0.9700
O5—H5A	0.79 (3)	C8—H8B	0.9700
C1—C2	1.384 (2)	C10—C11	1.503 (2)
C1—C6	1.396 (2)	C10—H10A	0.9700
C2—C3	1.377 (3)	C10—H10B	0.9700
			
C10—S1—C7	100.79 (8)	C6—C7—S2	113.81 (11)
C8—S2—C7	99.46 (8)	S1—C7—S2	109.04 (8)
C1—O1—H1	108.6 (19)	C6—C7—H7	109.2
C9—O2—H2A	112.0 (18)	S1—C7—H7	109.2
C11—O5—H5A	110.7 (19)	S2—C7—H7	109.2
O1—C1—C2	121.08 (15)	C9—C8—S2	115.36 (12)
O1—C1—C6	118.31 (15)	C9—C8—H8A	108.4
C2—C1—C6	120.61 (16)	S2—C8—H8A	108.4
C3—C2—C1	120.38 (17)	C9—C8—H8B	108.4
C3—C2—H2	119.8	S2—C8—H8B	108.4
C1—C2—H2	119.8	H8A—C8—H8B	107.5
C2—C3—C4	119.95 (17)	O3—C9—O2	124.33 (17)
C2—C3—H3	120.0	O3—C9—C8	125.59 (17)
C4—C3—H3	120.0	O2—C9—C8	110.08 (16)
C3—C4—C5	119.65 (18)	C11—C10—S1	115.58 (12)
C3—C4—H4	120.2	C11—C10—H10A	108.4
C5—C4—H4	120.2	S1—C10—H10A	108.4
C4—C5—C6	121.41 (17)	C11—C10—H10B	108.4
C4—C5—H5	119.3	S1—C10—H10B	108.4
C6—C5—H5	119.3	H10A—C10—H10B	107.4
C5—C6—C1	117.99 (15)	O4—C11—O5	123.98 (18)
C5—C6—C7	120.20 (15)	O4—C11—C10	125.68 (17)
C1—C6—C7	121.74 (14)	O5—C11—C10	110.34 (15)
C6—C7—S1	106.43 (11)		
			
O1—C1—C2—C3	178.98 (17)	C5—C6—C7—S2	51.35 (19)
C6—C1—C2—C3	−0.4 (3)	C1—C6—C7—S2	−131.74 (14)
C1—C2—C3—C4	0.0 (3)	C10—S1—C7—C6	−172.98 (11)
C2—C3—C4—C5	0.3 (3)	C10—S1—C7—S2	63.85 (9)
C3—C4—C5—C6	−0.3 (3)	C8—S2—C7—C6	88.30 (13)
C4—C5—C6—C1	−0.1 (3)	C8—S2—C7—S1	−153.06 (9)
C4—C5—C6—C7	176.96 (17)	C7—S2—C8—C9	69.48 (14)
O1—C1—C6—C5	−178.99 (16)	S2—C8—C9—O3	7.7 (2)
C2—C1—C6—C5	0.4 (2)	S2—C8—C9—O2	−171.86 (13)
O1—C1—C6—C7	4.0 (2)	C7—S1—C10—C11	77.55 (14)
C2—C1—C6—C7	−176.55 (15)	S1—C10—C11—O4	−2.3 (3)
C5—C6—C7—S1	−68.78 (18)	S1—C10—C11—O5	178.19 (13)
C1—C6—C7—S1	108.13 (15)		

### Hydrogen-bond geometry (Å, º)

**Table:**

*D*—H···*A*	*D*—H	H···*A*	*D*···*A*	*D*—H···*A*
C7—H7···O1	0.98	2.39	2.846 (2)	108
C2—H2···O3^i^	0.93	2.58	3.395 (2)	146
O1—H1···O4^ii^	0.75 (2)	1.97 (2)	2.7143 (19)	173 (3)
C8—H8*B*···O5^iii^	0.97	2.53	3.429 (2)	154
O2—H2*A*···O1^iv^	0.81 (3)	1.92 (3)	2.706 (2)	163 (2)
O5—H5*A*···O3^v^	0.79 (3)	1.97 (3)	2.7459 (19)	165 (3)

Symmetry codes: (i) *x*+1, *y*+1, *z*; (ii) −*x*+2, −*y*+1, −*z*; (iii) *x*, *y*+1, *z*; (iv) −*x*+1, −*y*+1, −*z*; (v) −*x*+1, −*y*, −*z*.
